# Tribological properties of nanolamellar tungsten disulfide doped with zinc oxide nanoparticles

**DOI:** 10.1186/s40064-015-1488-3

**Published:** 2015-11-04

**Authors:** V. An, Y. Irtegov, E. Anisimov, V. Druzyanova, N. Burtsev, M. Khaskelberg

**Affiliations:** Laboratory 12, Institute of High Technology Physics, National Research Tomsk Polytechnic University, 30 Lenin Ave., 634050 Tomsk, Russia; North-Eastern Federal University, Belinskogo 58, Yakutsk, Russia

**Keywords:** Tungsten disulfide, Nanoparticles, Friction coefficient

## Abstract

Tribological properties of nanolamellar tungsten disulfide doped with zinc oxide nanoparticles were studied. Nanolamellar tungsten disulfide and ZnO nanoparticles produced by electrospark erosion of metal granules in an H_2_O_2_ solution were analyzed using the XRD, SEM and TEM techniques. According to the tribological measurements, ZnO nanoparticles did not significantly change the friction coefficient of nanolamellar WS_2_ at 25 °C in air, whereas they positively impact on wear resistance of nanolamellar WS_2_ at 400 °C.

## Background

Tungsten disulfide doped with nanostructured zinc oxide is a promising solid lubricant which reveals excellent lubricant performance. Tribological behavior is related to changes at a high temperature (over 400 °C), when zinc oxide interacts with WO_3_ and forms ZnWO_4_ (zinc tungstate). ZnWO_4_ reveals higher thermal stability in air at a high temperature than pure WS_2_ and displays better lubricant performance than pure ZnO (Prasad et al. 2000). This fact was found for thin burnished films of WS_2_ micron-sized powder and ZnO nanopowder mixed in a 1:1 ratio.

Doping polymers with pure ZnO resulted in ambiguous changes in tribological performance. Wear can decrease with an increase in the zinc oxide concentration, meanwhile an increase of 20–30 % in the friction coefficient was reported (Wanga et al. 2009; Songa et al. 2010). Additives of zinc oxide nanoparticles can also impact on tribological properties of oil (Hernandez Battez et al. 2008). However, ZnO is considered as eco-neutral, stable in air at higher temperatures (>1000 °C) and can be exploited under extreme conditions.

Previous studies have shown excellent tribological performance of nanolamellar tungsten disulfide prepared by self-propagating high-temperature synthesis (SHS) from W nanopowders (Irtegov and An 2014; An et al. 2014; An and Irtegov 2014). The limitations in the WS_2_ application are related to its thermal stability in air (An et al. 2014). The present work is therefore aimed at studying tribological properties of nanolamellar tungsten disulfide doped with zinc oxide nanoparticles.

## Results and discussion

**Fig. 1:**
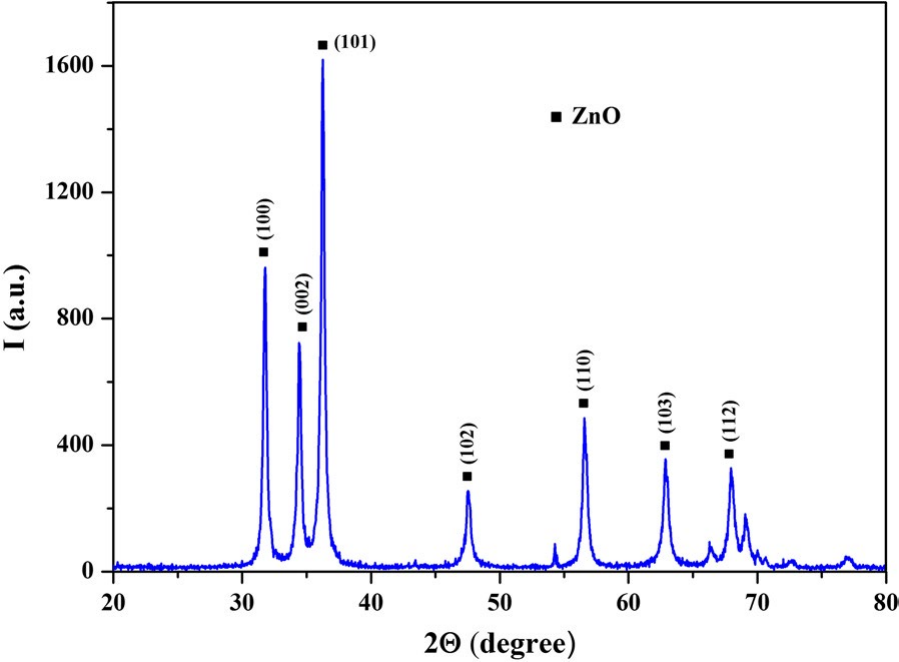
XRD pattern of ZnO nanoparticles synthesized by electrospark erosion

**Fig. 2:**
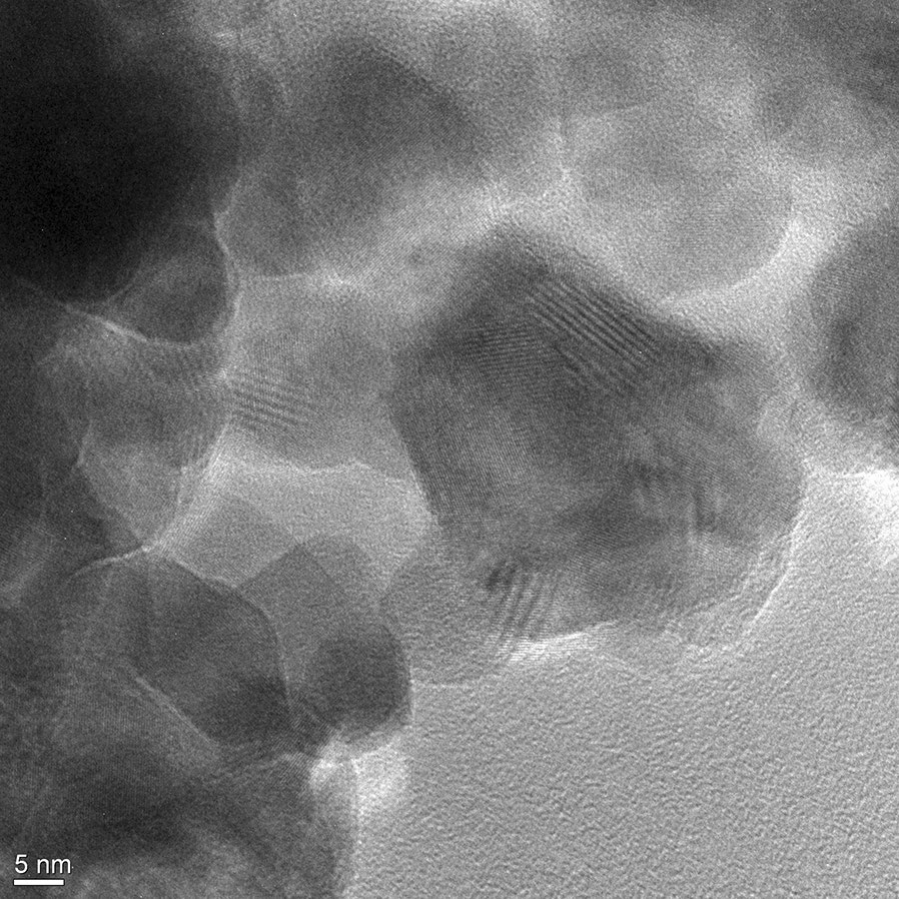
TEM of ZnO nanoparticles synthesized by electrospark erosion

**Fig. 3:**
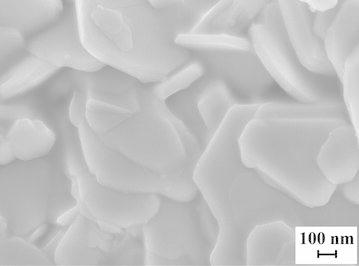
SEM image of nanolamellar WS_2_ produced by self-propagating high-temperature synthesis

**Fig. 4:**
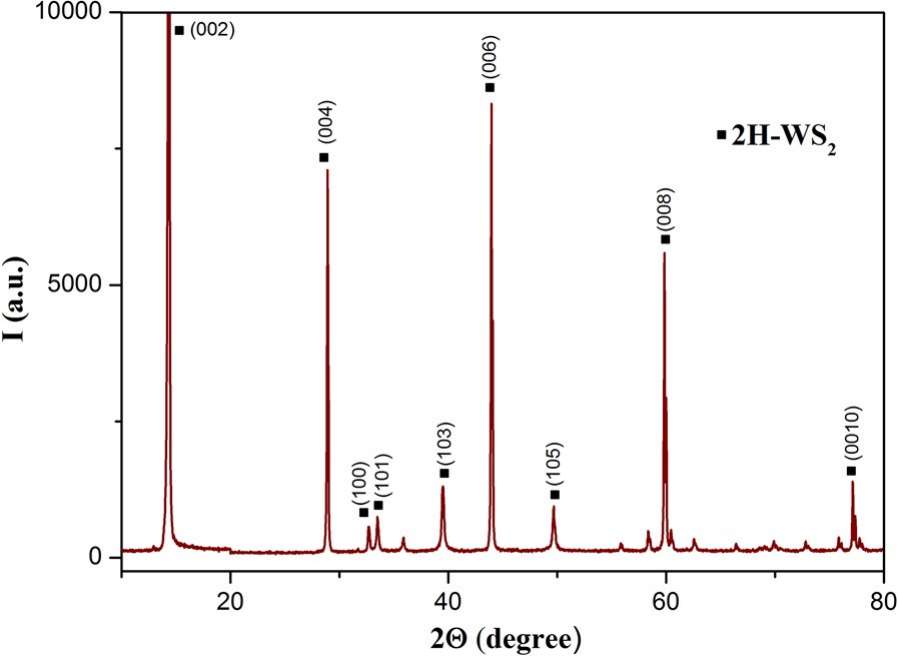
XRD pattern of nanolamellar WS_2_ produced by self-propagating high-temperature synthesis

The X-ray diffraction measurements (Fig. 1) show that the main phase of the powder prepared by electrospark erosion of zinc granules in an H_2_O_2_ solution is zinc oxide ZnO (zincite, PDF# 361451). The calculations according to the Scherrer’s formula demonstrate that the mean size of the ZnO crystallites is about 24 nm which corresponds well to the TEM observations (Fig. 2). The synthesized ZnO powder are hexagonal particles of 15–30 nm in width which form agglomerates of several microns in width. It is also in a good agreement with the XRD data showing that the main phase is hexagonal zinc oxide. The small size and the hexagonal structure of zinc oxide nanoparticles (n-ZnO) can play an important role in lubrication processes by filling microcracks of friction surfaces. As shown in Fig. 3, the as-prepared nanolamellar WS_2_ presented agglomerates of lamellar particles with a thickness of 50–150 nm. The particles were obviously well crystallized in hexagonal lattice what was confirmed by the XRD data (Fig. 4). The lamellas are 20–40 nm wide. Some lamellar particles possess multilayer structure (Fig. 3).

**Fig. 5:**
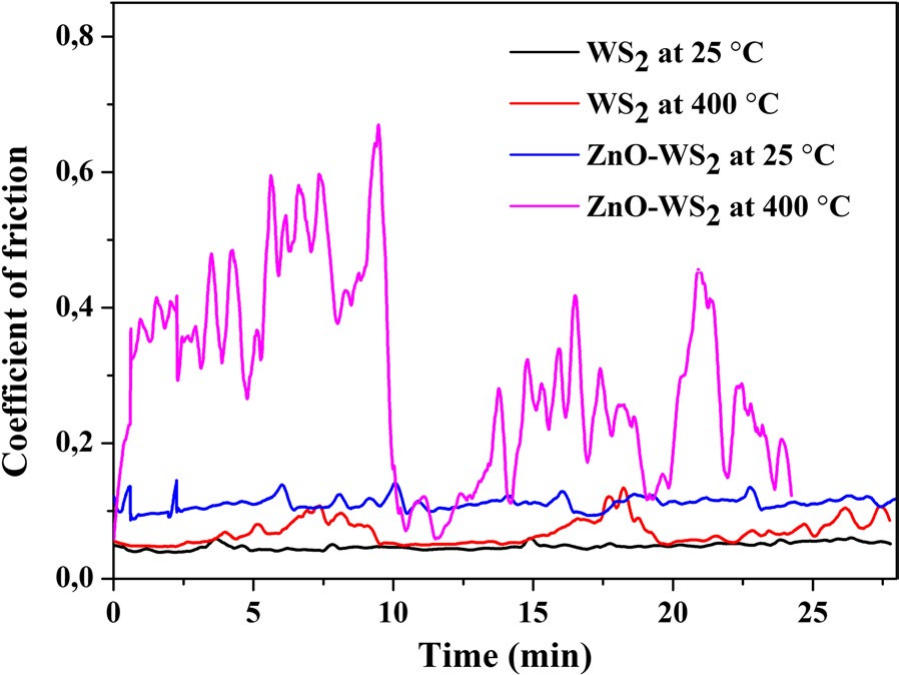
Friction coefficient versus time for undoped nanolamellar WS_2_ at 25 and 400 °C, nanolamellar WS_2_ doped with n-ZnO at 25 and 400 °C

The additive of ZnO nanoparticles in nanolamellar WS_2_ powder resulted in a low increase of the friction coefficient at 25 °C (Fig. 5) in comparison with the undoped powder. The observed effect can be explained by the difference in the hardness of zinc oxide and tungsten disulfide what results in indentation of ZnO nanoparticles in the metal disulfide nanolayer under friction according to the mechanism described in (Prasad et al. 2000). Thus, low friction of nanolamellar WS_2_ doped with n-ZnO at 25 °C is provided by nanolamellar tungsten disulfide. At 400 °C, the ZnO–WS_2_ composition exhibits an unstable friction coefficient (Fig. 5, rose curve) while the pure WS_2_ has a low and a more stable friction coefficient (Fig. 5, red curve). After 10 min of the test, reduction of the friction coefficient up to an average value µ = 0.23 was observed in comparison with the results obtained for burnished ZnO–WS_2_ films at 500 °C (Prasad et al. 2000). The friction coefficient fluctuations can be explained by the more intensive tribochemical transformation of tungsten disulfide into tungsten oxide with the following interaction with n-ZnO.

**Fig. 6:**
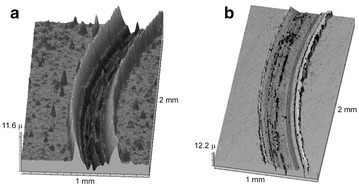
Wear tracks of the steel disk after the friction tests with undoped nanolamellar WS_2_ (**a**) and nanolamellar WS_2_ doped with n-ZnO (**b**) at 400 °C in air

Examination of the worn steel disk after the friction test at 400 °C showed a more visible effect of ZnO nanoparticles on the performance of nanolamellar WS_2_ (Fig. 6). We can see a decrease in the wear track depth and degradation of the steel disk surface for the nanolamellar WS_2_ doped with n-ZnO (Fig. 6a, b). Nevertheless, the wear track surface for this sample displays cavities which are caused by the use of zinc oxide.

## Conclusions

The additive of zinc oxide nanoparticles showed an insignificant increase in the friction coefficient of the composite lubricant and low friction was supplied by nanolamellar tungsten disulfide at 25 °C. The nanolamellar WS_2_ doped with n-ZnO showed ambiguous results in the tribological experiments in air at 400 °C which can be an object of additional studies. Apparently, doping nanolamellar WS_2_ with ZnO nanoparticles can lead to a positive effect on wear at high temperature.

## Experimental

ZnO nanoparticles were synthesized by electrospark erosion of zinc granules in an H_2_O_2_ solution (Galanov et al. 2013). A ceramic cylinder served as a synthesis reactor. The synthesis reactor was charged with about 100 g of zinc granules of 5 mm in diameter. Zinc electrodes were placed into the reactor which was then filled with 200 ml of 40 % H_2_O_2_. The electrodes were connected to a pulse current supply with the following characteristics: pulse duration—10 µs, pulse frequency—100 Hz, voltage—500 V, and first pulse half-cycle current—250 A. The obtained suspension was dried after the process at 60 °C in air.

Tungsten disulfide was synthesized via the method reported in the previous work (Irtegov et al. 2012). After drying, the synthesized powder was examined using the X-ray diffraction (Shimadzu XRD-7000 diffractometer, CuK_α_ radiation), SEM (JSM-7500FA, JEOL) and TEM (JEM-2100F, JEOL) techniques. The size of crystallites of as-prepared ZnO nanoparticles was calculated using the Scherrer formula:

$$d = \frac{9 \cdot \lambda }{\beta \cdot \cos \vartheta },$$

where λ is the is the X-ray wavelength, β is the line broadening at half the maximum intensity (FWHM), after subtracting the instrumental line broadening, θ is the Bragg angle.

Nanolamellar tungsten disulfide and zinc oxide nanoparticles (n-ZnO) were mechanically mixed in a 1:1 weight ratio. Tribological properties of the doped WS_2_ nanolamellar powder were then studied. The friction coefficient of nanolamellar WS_2_ doped with n-ZnO was measured with a “ball-on-disk” PC-Operated High Temperature Tribometer (THT-S-AX0000, CSEM). The worn surfaces were studied using a non-contact profilometer (Micro Measure 3D Station, STIL, France). Medium-carbon steel disks of diameter 30 mm, height 4 mm, and surface roughness Ra = 30–50 nm were used as the body. A 3 mm hard alloy ball was used as the counterbody. The normal load was 5 N, the temperature was 25 and 400 °C, the linear speed was 5 cm/s, and the wear scar radius was 3 mm.
